# Comparison of Different Combinations of Irradiation Mode and Jaw Width in Helical Tomotherapy for Nasopharyngeal Carcinoma

**DOI:** 10.3389/fonc.2020.00598

**Published:** 2020-04-23

**Authors:** Jun Zhang, Yinglin Peng, Shouliang Ding, Jinhan Zhu, Yimei Liu, Meining Chen, Wenzhao Sun, Linghong Zhou, Xiaowu Deng

**Affiliations:** ^1^Department of Radiation Oncology, Sun Yat-sen University Cancer Center, State Key Laboratory of Oncology in South China, Collaborative Innovation Center for Cancer Medicine, Guangdong Key Laboratory of Nasopharyngeal Carcinoma Diagnosis and Therapy, Guangzhou, China; ^2^School of Biomedical Engineering, Southern Medical University, Guangzhou, China; ^3^School of Biomedical Engineering, Sun Yat-sen University, Guangzhou, China

**Keywords:** helical tomotherapy, nasopharyngeal carcinoma, dynamic jaw, irradiation mode, jaw width

## Abstract

**Purpose:** To aid in the selection of a suitable combination of irradiation mode and jaw width in helical tomotherapy (HT) for the treatment of nasopharyngeal carcinoma (NPC).

**Materials and Methods:** Twenty patients with NPC who underwent radiotherapy were retrospectively selected. Four plans using a jaw width of 2.5 or 5-cm in dynamic jaw (DJ) or fix jaw (FJ) modes for irradiation were designed (2.5DJ, 2.5FJ, 5.0DJ, and 5.0FJ). The dose parameters of planning target volume (PTV) and organs at risk (OARs) of the plans were compared and analyzed, as well as the beam on time (BOT) and monitor unit (MU). The plans in each group were ranked by scoring the doses received by the OARs and the superity was assessed in combination with the planned BOT and MU.

**Results:** The prescribed dose coverage of PTV met the clinical requirements for all plans in the four groups. The groups using a 2.5-cm jaw width or a DJ mode provided better protection to most OARs, particularly for those at the longitudinal edges of the PTV (*P* < 0.05). The 2.5DJ group had the best ranking for OAR-dose, followed by the 2.5FJ and 5.0DJ groups with a same score. The BOT and MU of the groups using a 5.0-cm jaw width reduced nearly 45% comparing to those of the 2.5-cm jaw groups.

**Conclusion:** 2.5DJ has the best dose distribution, while 5.0DJ has satisfactory dose distribution and less BOT and MU that related to the leakage dose. Both 2.5DJ or 5DJ were recommended for HT treatment plan for NPC based on the center workload.

## Introduction

Helical tomotherapy (HT) utilizes the opening and closing of a 64-leaf, pneumatically powered, binary multileaf collimator with 51 equally-spaced beam angles at 360° and translational motion of the treatment couch at a constant speed to achieve a high degree of freedom and power in dose optimization (1). Since HT can attain superior conformity of the dose distribution and homogeneity of the target dose, it has been widely adopted as a radiotherapy modality for various malignant tumors (2–5). HT is particularly suitable for the treatment of patients with nasopharyngeal carcinoma (NPC), who have target volumes with complex shapes and numerous organs at risk (OARs) in the surrounding area (6, 7). Dose optimization for HT is achieved by adjusting specific parameters of the plan, primarily the modulation factor, jaw width, and pitch (1).

A radiotherapy plan in clinical practice should be selected considering not only the quality of dose distribution but also the delivery time and treatment efficiency, which is related to the patient motion and target displacement during treatment, as well as the total number of monitor units (MUs) associated with leakage dose received by the patient. In NPC, many OARs are present adjacent to the edges of the target volume in the longitudinal direction, such as the hippocampus, temporal lobe, and optic nerve. The use of a narrower jaw width for beam delivery can provide better conformity of dose distribution, which can better protect the aforementioned organs while markedly increasing beam-on time (BOT) and number of MUs. Conversely, when a wider jaw width is used for beam delivery, the BOT and number of MUs are greatly reduced; however, the conformity of dose distribution may be unsatisfactory. This may increase the doses received by normal organs, such as the hippocampus and temporal lobe. The hippocampus is an important organ for memory and cognitive function in humans. The control and reduction of radiation dose received by the hippocampus are critical for the quality of long-term survival in patients with NPC who achieve satisfactory therapeutic effects (8–10). Therefore, to ensure better protection of OARs, clinical planning of HT for NPC often involves using a jaw width of 2.5-cm and partly sacrifices the delivery efficiency when using the conventional fixed jaw mode.

Conventional HT utilizes the fixed jaw mode of the collimator for beam delivery. The collimator jaw of the machine is fully opened to the predetermined width as soon as one of the edges of the target volume in the longitudinal direction enters the beam and is only completely closed when the other edge of the target volume exits the beam. Therefore, the width of the penumbra of the target volume in the longitudinal direction completely depends on the selected jaw width (11), and using a wider jaw width in this fixed jaw mode will lead to a considerable dose of scatter radiation in the craniocaudal edges of the target volume. The recently released novel Precision tomotherapy platform provides a dynamic jaw (DJ) mode that can deliver a radiation beam dynamically to the superior and inferior borders of the target volume by using narrower jaw widths (12). Several studies have reported an improved HT efficiency for the treatment of thoracic, abdominal, total marrow, and prostate diseases using a combination of relatively large jaw width and DJ mode, followed by the dynamic reduction of jaw width at the edges of the target volume in the longitudinal direction. Moreover, the penumbras at the edges of the target volumes in the longitudinal direction are significantly reduced (13–17).

NPC is a common malignant tumor in China and Southeast Asia, with intensity-modulated radiation therapy being the primary treatment modality. Due to the complex shapes of the target volumes and the proximity to many important normal tissues and organs in NPC, the dose conformity requirement when using radiotherapy is extremely high. Thus, NPC is particularly suitable for HT. However, there are few reports on the application and parameter selection in tomotherapy using DJ technology for NPC. This study compared the effects of different irradiation modes and jaw widths in HT on the quality of dose distribution, delivery efficiency, and MUs for the treatment of NPC. The objective of this study was to provide a reference for the selection of a combination of irradiation mode and jaw width in HT suitable for the clinical treatment of NPC.

## Materials and Methods

### Patients' Clinical Characteristics

Twenty patients with NPC who underwent radiotherapy between 2017 and 2019 were retrospectively and randomly selected. All patients had pathologically confirmed, poorly differentiated squamous cell carcinoma. The 19 males and 1 female were 28–69 years of age, with a median age of 50.5 years.

### Image Acquisition at Simulation

All patients were placed in a supine position and a thermoplastic mask was fixed in place. Contrast-enhanced helical computed tomography (CT) scans (Somatom Sensation Open, Siemens AG) were then performed under the following conditions: 140 kV, 280 mAs, a scan and reconstruction slice thickness of 3-mm, and a pitch of 1:1. The scan range was from the top of the head to 2-cm below the clavicle. Each patient also underwent magnetic resonance imaging (MRI) (Ingenia 3.0T, Philips) using the same immobilization and the same scan range to acquire images for localization. MRI sequences, including T1, T2, contrast-enhanced T1, and fat-suppressed T1, were acquired. The CT and MR images acquired as described above were transmitted to the treatment planning system (Monaco version 5.1, Elekta) for target volume and OAR delineation.

### Delineations of Target Volumes and OARs

The target volumes and OARs for all patients were delineated by radiation oncologists based on the MRI and contrast-enhanced CT simulation images, in accordance with the ICRU50 (18) and ICRU62 reports (19), which included the gross tumor target volume in the nasopharynx (GTV_nx_), the nodal target volume in the neck (GTV_nd_), the high-risk clinical target volume (CTV_1_), and the preventive clinical target volume (CTV_2_). Following the delineations of the above target volumes, the corresponding planning target volumes (PTVs) were generated by the treatment planning system (TPS) through margin expansion to account for positioning errors and were defined correspondingly as PTV_nx_, PTV_nd_, PTV_1_, and PTV_2_, respectively. OARs were delineated based on the ICRU83 report (20) and primarily included the brain stem, spinal cord, lens, optic nerve, optic chiasm, hippocampus, pituitary, temporal lobe, inner ear, parotid gland, oral cavity, larynx, mandible, and temporomandibular joint. The corresponding planning organ at risk volumes were generated by the TPS through margin expansion to account for positioning errors.

### Plan Designs

The CT simulation images and contoured structures of each patient were transmitted to the treatment planning workstation (Precision 1.1.1.0; Accuray, Sunnyvale, CA, USA) for plan design. Four HT plans were designed for each patient: 2.5-cm-fixed jaw (2.5FJ), 2.5-cm-dynamic jaw (2.5DJ), 5.0-cm-fixed jaw (5.0FJ), and 5.0-cm-dynamic jaw (5.0DJ). All plans utilized a pitch of 0.287 and a modulation factor of 3.0. The dose calculation algorithm utilized convolution superposition and fine-matrix calculations were performed. The output dose rate was 1,180 MU/min. The prescribed doses to the target volumes were as follows: PTV_nx_, 70 Gy; PTV_nd_, 66 Gy; PTV_1_, 60 Gy; and PTV_2_, 54 Gy. All plans included 33 fractions and required that the prescribed dose coverage of the target volume be not <95% of the PTV and the maximum dose should not exceed 110% of the prescribed dose. All dose constraints for OARs were based on the ICRU83 report (20), the RTOG0615 protocol (21) and the international guidelines on dose prioritization and acceptance criteria in radiation therapy planning for nasopharyngeal carcinoma (22). Since there was a 30–40% of the parotid volume overlapped with the PTV2 in those cases of advanced staging. The center criteria of acceptance, mead dose of parotid gland <40 Gy (T3) and <45 Gy (T4) for advanced patients were used in the assessment and were verified by clinicians. All plans were optimized using the same dose constraints, and an identical and sufficient number of iterative optimizations were performed.

### Assessment of Plan Parameters

Dose volume histograms were used to assess the dose distributions in the target volumes and the dose volumes received by the OARs.

The parameters assessed for target volumes included the conformity index (CI) and heterogeneity index (HI) of the target volume, dose received by 98% of the target volume (D_98%_), percentage of target volume covered by the prescribed dose (V_100%_), maximum dose (D_max_), and mean dose (D_mean_). Since multiple dose gradients were used for NPC, the CI and HI were only calculated for PVT_nx_. The CI and HI were calculated using the following formula (23, 24):

$$\begin{array}{lll} CI & = & \frac{TV_{RI}}{TV}\times \frac{TV_{RI}}{V_{RI}}\\ HI & = & \left(D_{2\%}-D_{98\%}\right)/D_{p} \end{array}$$

where TV is the volume of the PTV (cm^3^), V_RI_ is the volume encompassed by the prescription isodose (cm^3^), TV_RI_ is the target volume covered by the prescription isodose (cm^3^); D_2%_ is the dose received by 2% of the volume of the PTV, D_98%_ is the dose received by 98% of the volume of the PTV, and D_P_ is the prescribed dose. A CI closer to 1 denotes better dose conformity of the target volume and a lower HI value indicates a more homogenous dose distribution within the target volume.

The parameters assessed for the OARs included the maximum dose to 1 cc volume (D_1cc_) of the brain stem, spinal cord, and temporal lobe; maximum dose (D_max_) to the brain stem, spinal cord, lens, optic nerve, optic chiasm, pituitary, and hippocampus; and the mean dose (D_mean_) to the optic nerve, optic chiasm, eyes, parotid gland, and hippocampus. The planned MUs and delivery time were recorded to evaluate the beam utilization and execution efficiency of the four plans.

### Statistical Analysis

Pairwise comparisons of dosimetric parameters in the four plans were analyzed using paired *t*-tests. All statistical analyses were performed using SPSS version 20.0 for Windows (IBM Corp., Armonk, NY, USA). Analysis items with *P* < 0.05 were considered statistically significant.

## Results

### Comparisons of Dosimetric Parameters of Target Volumes

**Figure 1:**
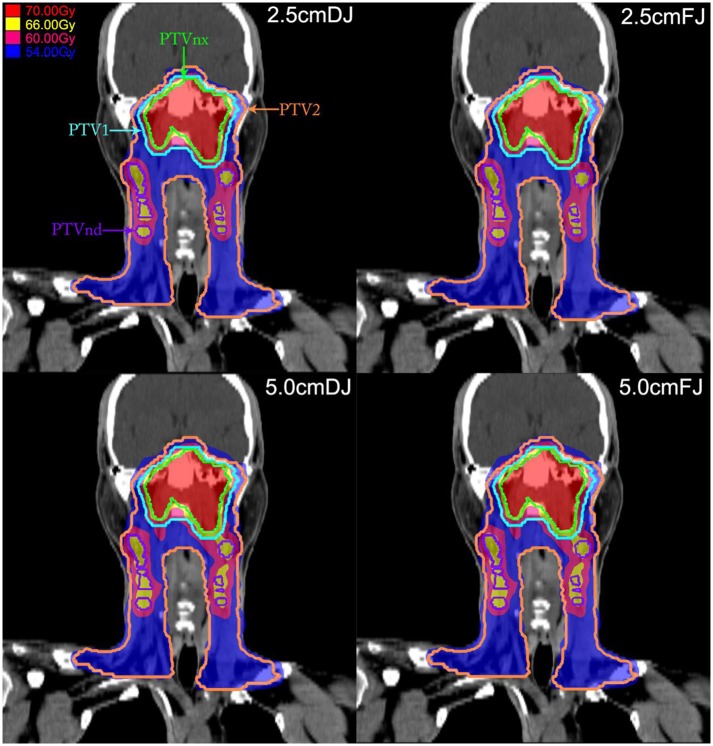
Dose distributions in the target volume in the coronal plane of the four plans in a patient with nasopharyngeal carcinoma (NPC). In these four plans, the 70-Gy dose cloud covers the planning target volume (PTV)_nx_ (green line), the 66-Gy dose cloud covers the PTV_nd_ (purple line), the 60-Gy dose cloud covers the PTV_1_ (cyan line), and the 54-Gy dose cloud covers the PTV_2_ (orange line).

All the four groups of treatment plan met the requirement of a 95% prescribed dose coverage of the target volume. The target dose CI showed greater differences between the 2.5-cm and 5-cm jaw width groups, irrespective of the FJ or DJ irradiation mode. The CI of the 2.5-cm jaw width groups were significantly better than those in the 5.0-cm jaw groups (*P* < 0.05). However, apart from the HI of the PTV_nx_ which was slightly better in the 2.5-cm jaw width groups, the differences in the remaining dosimetric parameters of target volumes in the four plans, including the V_100%_, D_98%_, D_max_, and D_mean_, were <1% among the groups. Since such minor differences did not have practical clinical significance, the target doses of the plans in the four groups were all acceptable. The comparisons of the dosimetric parameters of the target volumes in each group are shown in Table 1. Figure 1 shows the dose distributions in the coronal plane of the four plans for one patient.

**Table 1:** Comparisons of dosimetric parameters of target volumes among the four plans in 20 patients.

**PTVs**	**Parameters**	**2.5FJ**	**2.5DJ**	**5.0FJ**	**5.0DJ**	***P*^**2.5**^**	***P*^**5.0**^**	***P*^**F**^**	***P*^**D**^**	P*
PTV_nx_	D_98%_ (Gy)	69.99 ± 0.19	69.99 ± 0.17	70.00 ± 0.24	69.99 ± 0.23	0.453	0.47	0.711	0.899	0.766
	V_100%_ (%)	97.96 ± 1.03	97.92 ± 1.00	97.94 ± 1.09	97.90 ± 1.08	0.176	0.494	0.706	0.688	0.263
	D_mean_ (Gy)	71.66 ± 0.35	71.65 ± 0.35	72.00 ± 0.50	71.99 ± 0.45	0.043	0.635	0	0	0
	D_max_ (Gy)	74.32 ± 1.15	74.31 ± 1.15	74.54 ± 0.95	74.58 ± 0.86	0.624	0.352	0.227	0.156	0.165
	CI	0.91 ± 0.02	0.91 ± 0.02	0.86 ± 0.04	0.86 ± 0.03	1	0.891	0	0	0
	HI	0.042 ± 0.009	0.042 ± 0.009	0.050 ± 0.012	0.050 ± 0.012	0.297	0.577	0	0	0
PTV_nd_	D_98%_ (Gy)	66.11 ± 0.25	66.09 ± 0.26	65.96 ± 0.27	65.97 ± 0.23	0.119	0.947	0.011	0.012	0.003
	D_mean_ (Gy)	66.77 ± 0.25	66.76 ± 0.25	67.98 ± 0.32	67.99 ± 0.29	0.012	0.696	0.005	0	0
	D_max_ (Gy)	70.74 ± 1.17	70.72 ± 1.17	71.13 ± 1.31	71.17 ± 1.27	0.032	0.53	0.008	0	0.001
PTV_1_	D_98%_ (Gy)	60.39 ± 0.55	60.36 ± 0.51	60.99 ± 0.86	60.99 ± 0.83	0.056	0.904	0	0	0
	V_100%_ (%)	98.44 ± 1.27	98.41 ± 1.18	99.00 ± 1.02	99.00 ± 1.05	0.467	0.881	0	0	0
PTV_2_	D_98%_ (Gy)	54.13 ± 0.42	54.13 ± 0.40	54.01 ± 0.62	53.99 ± 0.58	0.789	0.33	0.068	0.029	0.02
	V_100%_ (%)	98.20 ± 1.09	98.16 ± 1.14	97.74 ± 1.31	97.80 ± 1.21	0.088	0.419	0.001	0	0

P^2.5^: 2.5FJ vs. 2.5DJ; P^5.0^: 5.0FJ vs. 5.0DJ; P^F^: 2.5FJ vs. 5.0FJ; P^D^: 2.5DJ vs. 5.0DJ; P^*^: 2.5FJ vs. 5.0DJ.

### Comparisons of Dosimetric Parameters of OARs

Based on the same jaw width, the doses received by OARs in the DJ groups were all lower than or equivalent to those in the FJ groups. In the DJ groups, the doses received by OARs adjacent to the edges of the target volumes in the longitudinal (craniocaudal) direction, including the D_max_ and D_mean_ of the optic nerve, optic chiasm, and pituitary gland, D_mean_ of the eyes and temporal lobe, and D_1cc_ of the hippocampus, were all significantly lower than those in the FJ groups with the same jaw width (*P* < 0.05). No significant differences were observed for the other dosimetric parameters of the OARs between the DJ and FJ groups (*P* > 0.05).

**Figure 2:**
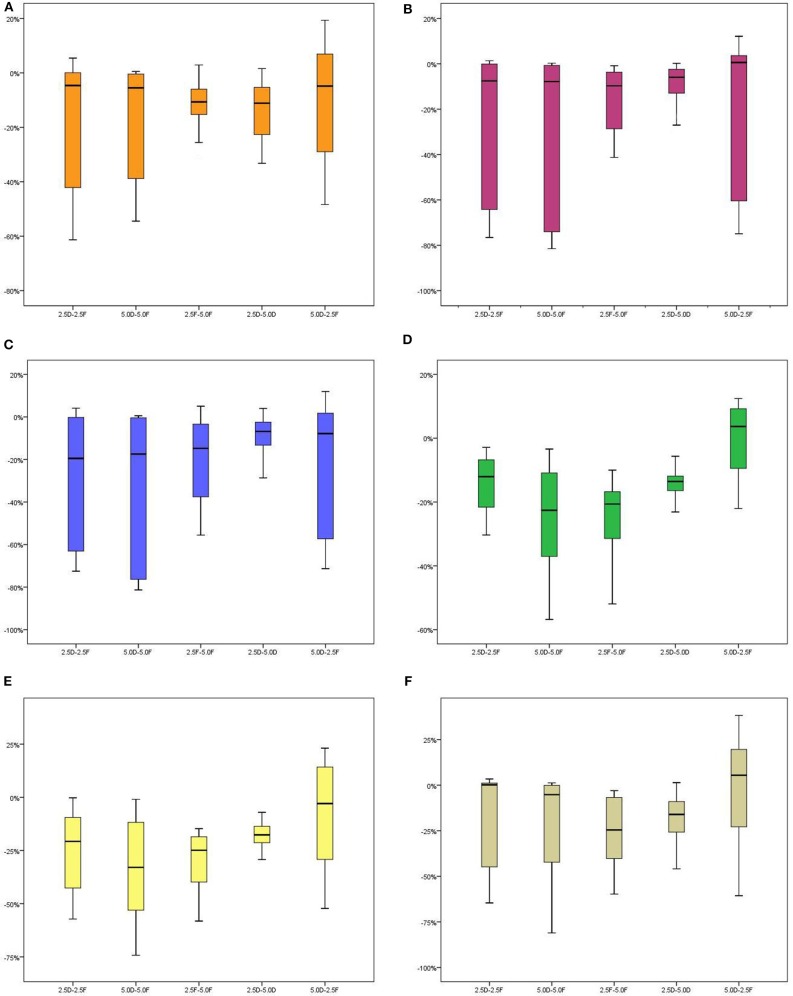
Stem-and-leaf displays of percentage differences in dosimetric parameters of several OARs among the four plans in 20 patients. Percentage difference $=\frac{X_{i}-X_{j}}{X_{i}}\times 100\%$. For example, the percentage difference between the 2.5DJ and 2.5FJ groups was calculated as the difference between the 2.5DJ dose minus the 2.5FJ dose, divided by the 2.5DJ dose, multiplied by 100%. [**(A)** D_mean_ of optic nerves, **(B)** D_max_ of optic chiasma, **(C)** D_mean_ of optic chiasma, **(D)** D_mean_ of temporal lobes, **(E)** D_mean_ of hippocampus, **(F)** D_1cc_ of hippocampus].

Based on the same irradiation mode, the doses received by almost all OARs in the 2.5-cm jaw width groups were lower than those in the 5.0-cm jaw width groups (*P* < 0.05). The groups using a narrow jaw width showed better OAR protection, particularly for OARs adjacent to the edges of the target volumes in the longitudinal direction. In the FJ mode, the D_mean_ of the optic nerve, D_max_ and D_mean_ of the optic chiasm, D_mean_ of the temporal lobe, and D_1cc_ and D_mean_ of the hippocampus were significantly lower in the 2.5FJ group than in the 5.0FJ group, with respective reductions of 11.04, 15.93, 20.44, 24.20, 25.40, and 30.15%. In the DJ mode, the D_mean_ of the optic nerve, D_mean_ of the temporal lobe, D_1cc_ and D_mean_ of the hippocampus in the 2.5DJ group were reduced by 12.92, 13.87, 17.09, and 17.47%, respectively, compared to those in the 5.0DJ group (Table 2 and Figure 2).

**Table 2:** Comparisons of dosimetric parameters of OARs among the 4 HT plans in 20 patients.

**OARs**	**Parameters**	**2.5FJ**	**2.5DJ**	**5.0FJ**	**5.0DJ**	***P*^**2.5**^**	***P*^**5.0**^**	***P*^**F**^**	***P*^**D**^**	P*
Spinal cord	D_max_ (Gy)	38.20 ± 6.06	38.22 ± 6.05	38.19 ± 5.77	38.22 ± 5.75	0.436	0.51	0.91	0.978	0.888
	D_1cc_ (Gy)	34.50 ± 1.87	34.51 ± 2.16	33.78 ± 2.16	33.82 ± 2.15	0.891	0.277	0.001	0.002	0.002
Brain stem	D_max_ (Gy)	60.42 ± 6.54	60.66 ± 6.27	61.82 ± 5.60	61.94 ± 5.49	0.033	0.117	0.01	0.016	0.006
	D_1cc_ (Gy)	53.93 ± 5.65	54.11 ± 5.48	55.45 ± 5.81	55.56 ± 5.66	0.012	0.178	0	0	0
Lens	D_max_ (Gy)	6.10 ± 1.01	6.06 ± 1.03	6.39 ± 1.04	6.33 ± 1.04	0.443	0.157	0.003	0.003	0.025
Optic nerve	D_max_ (Gy)	49.69 ± 10.46	43.76 ± 17.00	52.29 ± 9.29	46.82 ± 15.08	0.002	0.002	0.002	0	0.052
	D_mean_ (Gy)	33.77 ± 9.00	28.06 ± 14.33	37.58 ± 7.25	30.91 ± 13.18	0.001	0.001	0	0	0.046
Optic chiasm	D_max_ (Gy)	44.02 ± 15.66	35.69 ± 24.21	51.04 ± 11.91	38.35 ± 24.82	0.001	0.001	0	0	0.017
	D_mean_ (Gy)	38.54 ± 16.34	31.38 ± 22.99	46.56 ± 11.06	33.36 ± 23.27	0	0	0	0.002	0.005
Eyes	D_mean_ (Gy)	9.96 ± 2.46	9.26 ± 2.87	10.83 ± 2.61	9.73 ± 3.21	0	0	0	0.003	0.351
Parotid	D_mean_ (Gy)	39.56 ± 2.87	39.53 ± 2.88	40.01 ± 3.05	39.99 ± 3.04	0.033	0.43	0	0	0
Pituitary	D_max_ (Gy)	51.84 ± 13.35	48.75 ± 16.87	56.30 ± 1.00	51.73 ± 15.29	0.003	0.006	0	0.001	0.919
	D_mean_ (Gy)	47.75 ± 14.11	43.32 ± 18.96	53.22 ± 10.19	46.23 ± 17.56	0.001	0.002	0	0	0.162
Temporal lobe	D_1cc_ (Gy)	62.99 ± 6.99	62.98 ± 6.99	65.16 ± 5.52	65.13 ± 5.55	0.68	0.567	0	0	0
	D_mean_ (Gy)	18.67 ± 5.41	16.31 ± 6.25	24.43 ± 5.16	18.91 ± 7.02	0	0	0	0	0.61
	V_60Gy_ (%)	1.96 ± 2.39	1.95 ± 2.39	2.92 ± 3.05	2.88 ± 3.02	0.163	0.069	0	0	0
Hippocampus	D_max_ (Gy)	49.06 ± 13.54	46.70 ± 17.00	53.60 ± 10.13	49.28 ± 16.25	0.066	0.02	0.001	0	0.852
	D_1cc_ (Gy)	29.45 ± 13.38	26.41 ± 16.25	37.57 ± 10.66	31.31 ± 17.56	0.007	0.002	0	0	0.237
	D_mean_ (Gy)	17.97 ± 6.42	14.60 ± 8.02	25.15 ± 5.38	17.75 ± 9.73	0	0	0	0	0.796

P^2.5^: 2.5FJ vs. 2.5DJ; P^5.0^: 5.0FJ vs. 5.0DJ; P^F^: 2.5FJ vs. 5.0FJ; P^D^: 2.5DJ vs. 5.0DJ; P^*^: 2.5FJ vs. 5.0DJ.

#### All Tested Plans Passed the Acceptance Criteria

To better assess the quality of dose distributions in the four plans, we rounded the mean values of the dosimetric parameters of all OARs in each plan and ranked each parameter in ascending order by dose values. Then, we assigned a score according to the ranking: the group with the lowest dose was ranked first and was assigned a corresponding score of 1, while the group with the highest dose was ranked fourth and assigned a score of 4. Two groups with identical dose values were assigned the same rank and the same score (e.g., if they were both ranked first, they would both be assigned a score of 1). Finally, the scores of each group were calculated, with the group with the lowest score considered the best. The ranking of the overall scores showed that among the four groups, the 2.5DJ group had the best overall score, followed by the 2.5FJ and 5.0DJ groups with the same score and the 5.0FJ group with the worst score (Tables 3, 4).

**Table 3:** Mean values of dosimetric parameters of organs at risk (OARs) among the four plans in 20 patients (rounded).

**OARs**	**Parameters**	**2.5FJ**	**2.5DJ**	**5.0FJ**	**5.0DJ**
Spinal cord	D_max_ (Gy)	38	38	38	38
	D_1cc_ (Gy)	35	35	34	34
Brainstem	D_max_ (Gy)	60	61	62	62
	D_1cc_ (Gy)	54	54	55	55
Lens	D_max_ (Gy)	6	6	6	6
Optic nerve	D_max_ (Gy)	50	44	52	47
	D_mean_ (Gy)	34	28	38	31
Optic chiasm	D_max_ (Gy)	44	36	51	38
	D_mean_ (Gy)	39	31	47	33
Eyes	D_mean_ (Gy)	10	9	11	10
Parotid	D_mean_ (Gy)	40	40	40	40
Pituitary	D_max_ (Gy)	52	49	56	52
	D_mean_ (Gy)	48	43	53	46
Temporal lobe	D_1cc_ (Gy)	63	63	65	65
	D_mean_ (Gy)	19	16	24	19
	V_60Gy_ (%)	2	2	3	3
Hippocampus	D_max_ (Gy)	49	47	54	49
	D_1cc_ (Gy)	29	26	38	31
	D_mean_ (Gy)	18	15	25	18

**Table 4:** Ranks and scores based on the mean values of dosimetric parameters of organs at risk (OARs) among the four plans in 20 patients.

**OARs**	**Parameters**	**2.5FJ**	**2.5DJ**	**5.0FJ**	**5.0DJ**
Spinal cord	D_max_ (Gy)	1	1	1	1
	D_1cc_ (Gy)	2	2	1	1
Brainstem	D_max_ (Gy)	1	2	3	3
	D_1cc_ (Gy)	1	1	2	2
Lens	D_max_ (Gy)	1	1	1	1
Optic nerve	D_max_ (Gy)	3	1	4	2
	D_mean_ (Gy)	3	1	4	2
Optic chiasm	D_max_ (Gy)	3	1	4	2
	D_mean_ (Gy)	3	1	4	2
Eyes	D_mean_ (Gy)	2	1	3	2
Parotid	D_mean_ (Gy)	1	1	1	1
Pituitary	D_max_ (Gy)	2	1	3	2
	D_mean_ (Gy)	3	1	4	2
Temporal lobe	D_1cc_ (Gy)	1	1	2	2
	D_mean_ (Gy)	2	1	3	2
	V_60Gy_ (%)	1	1	2	2
Hippocampus	D_max_ (Gy)	2	1	3	2
	D_1cc_ (Gy)	2	1	4	3
	D_mean_ (Gy)	2	1	3	2
Total score		36	21	52	36
Overall ranking		2	1	3	2

### Comparisons of BOTs and MUs

The delivery time (BOT) and MU for each plan was based on the calculation of the treatment planning system. Since the dose rate of HT was fixed, the proportions of the differences in the mean BOTs and numbers of MUs between the FJ and DJ groups were also the same. The BOT and MU values of the FJ and DJ modes were more similar when the jaw width was the same. However, regardless of irradiation mode, the BOT and MU were reduced by ~45% for a 5.0-cm jaw width compared to those for a 2.5-cm jaw width (Table 5).

**Table 5:** Mean beam-on time(s) (BOTs) and monitor units (MUs) in each plan group.

**Group**	**2.5FJ**	**2.5DJ**	**5.0FJ**	**5.0DJ**
Time (s)	389	390	215	216
Monitor units (MUs)	7,644	7,671	4,237	4,247

## Discussion

Precision, a novel tomotherapy technology platform, offers a DJ mode for beam delivery, which effectively improves the issue of scatter radiation doses at the edges of the irradiation field (25). This study compared the differences in dosimetric parameters for four plans with different combinations of jaw width and irradiation mode, including 2.5FJ, 2.5DJ, 5.0FJ, and 5.0DJ, in the treatment of NPC. The effects of the different combinations on dose distribution quality, delivery efficiency, and MU of HT plans for NPC treatment were analyzed. Furthermore, an overall score was assigned to the four combinations for superiority evaluation. The optimization algorithm of the Precision planning system prioritizes target doses. The target dose requirement must first be satisfied before the doses to OARs could be optimized based on predetermined constraints. This study repeatedly optimized the plans used for the same patient using the same optimization parameters. The results obtained from sufficient iterative optimizations of the plans were stable, which ensured the comparability of the four plans assessed in this study.

The results of this study showed that all four combinations of jaw width and irradiation mode for HT treatment of NPC met the prescribed dose coverage of the target volume requirement. The mean differences in dosimetric parameters of the target volumes among the plans were <1%. Such minor differences could be ignored during actual treatment in clinical practice. The comparison of the doses received by the OARs showed that a narrower jaw width or a DJ mode provided better protection to most OARs, particularly those at the edges of the target volumes in the longitudinal direction, including normal tissues such as the optic nerve, pituitary gland, temporal lobe, and hippocampus. Treatment using a wider jaw width in the FJ mode may result in out-of-field doses in extended regions due to the larger penumbra and higher doses of scatter radiation. This would, in turn, increase the out-of-field irradiated volume. Thus, the doses received and irradiated volumes of organs and tissues adjacent to the edges of the target volume in the longitudinal direction also increase (26–28). When a narrow jaw width was used in the FJ mode or when a narrow jaw opening was used at the edges of the target volume in the longitudinal direction in the DJ mode, a steeper dose gradient was formed outside the borders of the target volumes along the Y-axis. This overcame the pitfall of dose extension in the longitudinal direction caused by a wide jaw width in the FJ mode (28).

In NPC, many important OARs are adjacent to the edges of the target volumes in the longitudinal direction. Zeng et al. (10) established a clinical dose association model and performed an analysis using the equivalent dose in 2-Gy fractions (EQD2), reporting a biologically-equivalent tolerance dose of D_1cc_ for the development of temporal lobe injury of 62.83 Gy. Hsiao et al. (9) reported that patients with NPC showed significant cognitive decline after receiving radiotherapy when the D_mean_ of the temporal lobe was >36 Gy. The results of the present study indicated that reducing the jaw width or using the DJ mode for beam delivery significantly reduced the D_1cc_ of the temporal lobe while also significantly reducing the D_mean_ of the temporal lobe. Furthermore, Gondi et al. (29) demonstrated that an EQD2 to 40% of the bilateral hippocampi >7.3 Gy was associated with long-term impairment in list-learning and delayed recall. The anatomic location of the hippocampus is adjacent to or overlaps with the target volume of NPC in the longitudinal direction. Therefore, the hippocampus can be selectively protected during HT planning. The results of the present study showed mean doses to the hippocampus in the 2.5FJ, 2.5DJ, 5.0FJ, and 5.0DJ groups of 17.97 ± 6.42, 14.60 ± 8.02, 25.15 ± 5.38, and 17.75 ± 9.73 Gy, respectively. The reduction in jaw width or use of the DJ mode could effectively reduce the mean doses to the hippocampus. Due to the numerous organs surrounding the target volume in NPC, it is necessary to consider the doses received by the target volume and OARs collectively during treatment planning or plan selection. Therefore, in this study, we comprehensively evaluated the quality of the four plans with different combinations by ranking and assigning scores to each based on the doses received by the OARs. The results showed that the 2.5DJ group had the best (lowest) overall score, followed by the 2.5FJ and 5.0DJ groups, with the same scores, the 5.0FJ group with the worst score. Furthermore, regardless of irradiation mode, the BOT and MU were reduced by ~45% when a 5.0-cm jaw width was used compared to those for a 2.5-cm jaw width. Although the 2.5DJ group exhibited the best dose distribution, the delivery time and MU were 45% higher than those in the 5.0DJ group. An increase in delivery time implies an increased probability of patient position and target displacements during treatment, while an increased number of MUs results in increased leakage radiation, thereby increasing the risk of second primary malignancies (SPMs) (30). The incidence of SPM has always been underestimated in the past, mainly because most patients have a relatively short life expectancy after treatment or because the follow-up period was <15 years. With the possibility of longer-term survival and longer follow-up times, the incidence of SPM among patients who have undergone radiation therapy can be up to 20%. The time between radiotherapy and SPM may be at least 10 years and even up to 50 years in some cases (31). Since NPC is a tumor that can almost be completely cured and is associated with long-term survival (32), the issue of SPM in patients with cannot be ignored. The present study considered the quality of dose distribution, BOT, and number of MUs in the four plans, and concluded that both the 2.5 DJ and 5.0DJ group provided an excellent combination for use as an HT plan for NPC.

In summary, the use of a narrower jaw width or DJ mode in HT plans for NPC can provide better protection for OARs. The 5-cm jaw width can reduce the delivery time and number of MUs by 45%. We recommend both the 2.5DJ and 5.0DJ mode for the clinical HT treatment of NPC patients based on center work load. Although the 2.5DJ plan perform the best in the test, the 5.0DJ mode can be chosen as an alternative selection when considering the balance of treatment efficiency and plan quality or concern of the out-of-field dose (related to delivery MUs) for those patients with expected long-term survival. According to reported literatures, out-of-field radiation doses to normal tissues may be associated with an increased risk of secondary malignancies, particularly in long-term survivors.

In this study, for simplifying the effect to plan quality and focus on the combination of jaw width and delivery mode, we simply chose the fix pitch of 0.287 and consistent modulation factor of 3. However, these parameters should be chosen based on the size, length or shape of the PTV to get an optimal dose distribution. This might bring bios in real clinical application, and need further study on particular classified group testing.

## Data Availability Statement

The datasets of this research are backed up on the Research Data Deposit (RDD, https://www.researchdata.org.cn, approval number: RDDA2019001300) and are available on reasonable request.

## Ethics Statement

Our study was reviewed and approved by the IRB committee of Sun Yat-sen University Cancer Center, with the approval number of YB2018-40. As this study is only a retrospective analysis, informed consent exemption was approved (shown in the attached IRB document.

## Author Contributions

XD and LZ contributed conception and design of the study. JZha, SD, JZhu, and YL organized the database. JZha, YP, MC, and WS performed the statistical analysis. JZha wrote the first draft of the manuscript. JZha and YP wrote sections of the manuscript. All authors contributed to manuscript revision, read, and approved the submitted version.

## Conflict of Interest

The authors declare that the research was conducted in the absence of any commercial or financial relationships that could be construed as a potential conflict of interest.
